# Vestibular Schwannoma: Spontaneous tumor involution

**DOI:** 10.1016/S1808-8694(15)31189-7

**Published:** 2015-10-19

**Authors:** Norma de Oliveira Penido, Rodrigo P. Tangerina, Eduardo Macoto Kosugi, Carlos Eduardo Cesário de Abreu, Matheus Brandão Vasco

**Affiliations:** 1 PhD in Medicine. Affiliated Professor - Unifesp-Epm; 2 M. S. in Sciences - UNIFESP/EPM; 3 M.S. Student - UNIFESP/EPM; 4 M.S. in sciences - UNIFESP/EPM; 5 Medical Student - PIBIC

**Keywords:** acoustic neuromas, vestibular schwannoma, treatment

## Abstract

The natural history of Vestibular Schwannomas (VS) is yet not totally known, but most of them have the tendency to slow growth, sometimes without any kind of symptoms during the individual's entire time. About 69% of diagnosed VS do not grow at all and 16% of these can even regress. Considering tumors that grow, about 70% have grown less than 2mm an year. Advanced radiological diagnosis, especially magnetic resonance imaging with gadolinium helps us diagnose small and less symptomatic tumors. Treatment of choice still is complete tumor resection. Surgical approaches have improved considerably and have helped preserve facial nerve function and hearing. Considering VS's natural history, there is a possibility for conservative treatment for these tumors, because their growth in the first year after diagnosis predicts tumor growth behavior in the next years. Surgery should be done in cases of tumor growth, patient's desire or symptoms worsening. Moreover, in terms of postoperative sequelae, there is no difference between patients who underwent surgery immediately after diagnosis and those who underwent initial conservative treatment for these tumors.

## INTRODUCTION

The last decades have seen substantial changes in how vestibular schwannomas (VS) are approached, thanks to further knowledge acquired in regards of their natural history associated with an improvement in diagnostic methods and treatment techniques

Most of the times, VS is a benign slow growing tumor, and it may remain asymptomatic throughout the patient's life, diagnosed only during autopsies^1^.

The major development in image exams, especially gadolinium-contrasted MRI, led to an increase in the number of VS diagnosed, especially the small ones with minimal symptoms, which were missed in the past.

Moreover, there was a major development in VS treatment, with improvements in surgical approaches together with the use of the surgical microscope, and the pioneer in it was Dr. William House in 1961, which caused a marked reduction in the mortality associated with this surgical procedure, down to levels below 1%. With that, surgery for VS, which was described in the beginning of the previous century, but carefully considered because it bears high morbi-mortality rates^2^, became the treatment of choice for most patients.

Due to the progress in microsurgical techniques, the goal of surgery is no longer complete tumor removal, but also hearing and facial nerve preservation. However, these goals are not always achieved after surgical procedure, and it may cause hearing loss and some degree of facial paresis.

Based on these data, we see the need for a conservative approach in selected patients with VS. We hereby report on two cases in which there has been tumor regression during the observation period.

## CLINICAL CASES

### Case 1

OV, 48 years, male, white.

**Figure 1:**
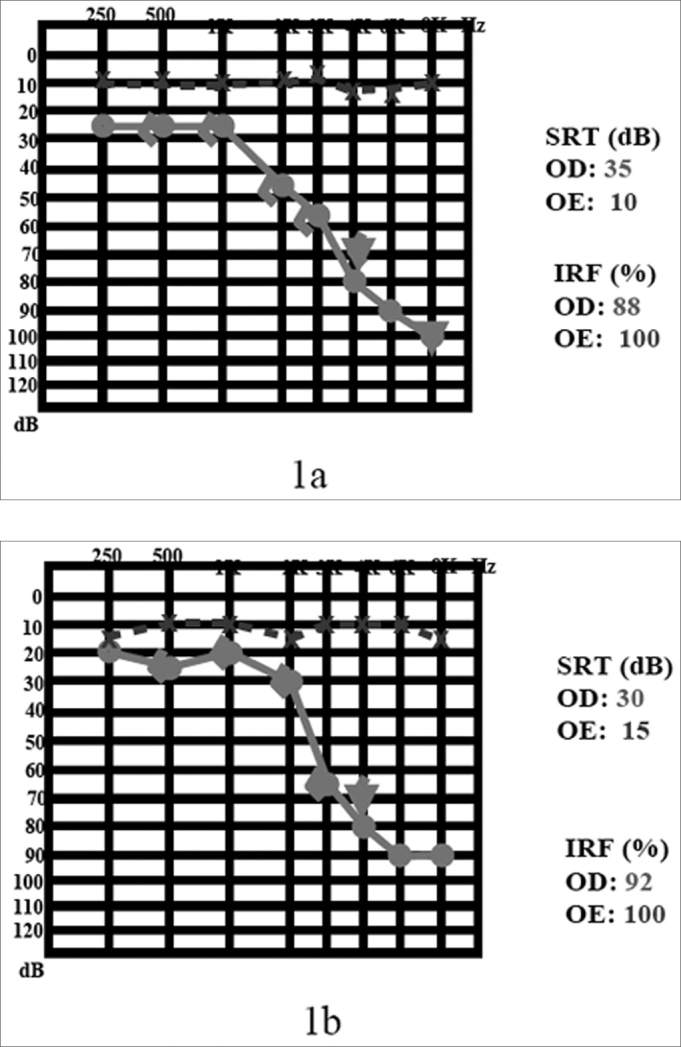
1a) audiogram at the time of diagnosis; 1b) after 4 years of follow up.

**Figure 2:**
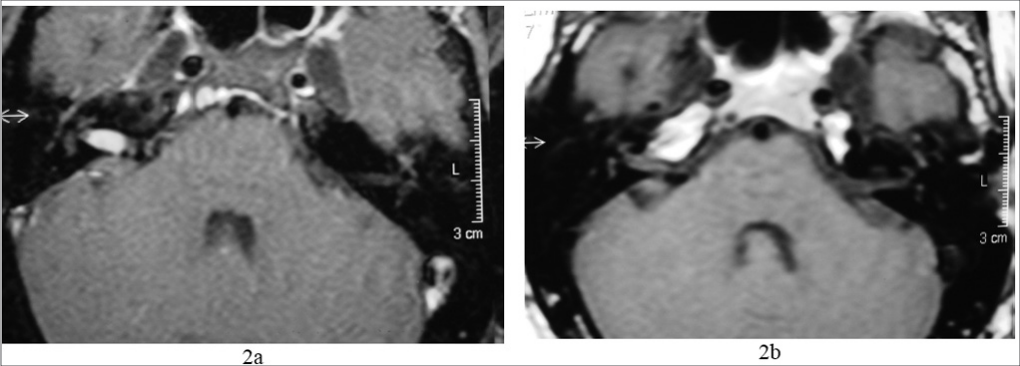
2a) MRI with gadolinium at the time of diagnosis; 2b) 4 years later, notice tumor involution.

Came to us complaining of tinnitus and hearing loss in his right ear for one year. He had normal physical exam. His audiogram showed a sensorineural hearing loss in the high frequencies of his right ear (Figure 1a). MRI showed a mass in his right inner acoustic meatus, measuring 1.0 × 0.6 × 0.6 (Figure 2a).

After explaining the risks and the benefits associated with VS treatment, the patient chose a conservative approach, with follow up by audiograms and MRI in series.

After 1 year of follow up, the patient reported a worsening in his tinnitus. Control audiogram showed a worsening in his hearing in the frequencies of 4, 6 and 8 kHz. Control MRI did not show changes in tumor size.

With patient participation we decided to keep the conservative approach.

The patient has been stable for 4 years, with improvement in his hearing thresholds in the frequencies of 2 and 3 kHz (Figure 1b), and a most recent MRI showed an important spontaneous tumor reduction (Figure 2b).

### Case 2

MNLC, 60 years, female, white.

**Figure 3:**
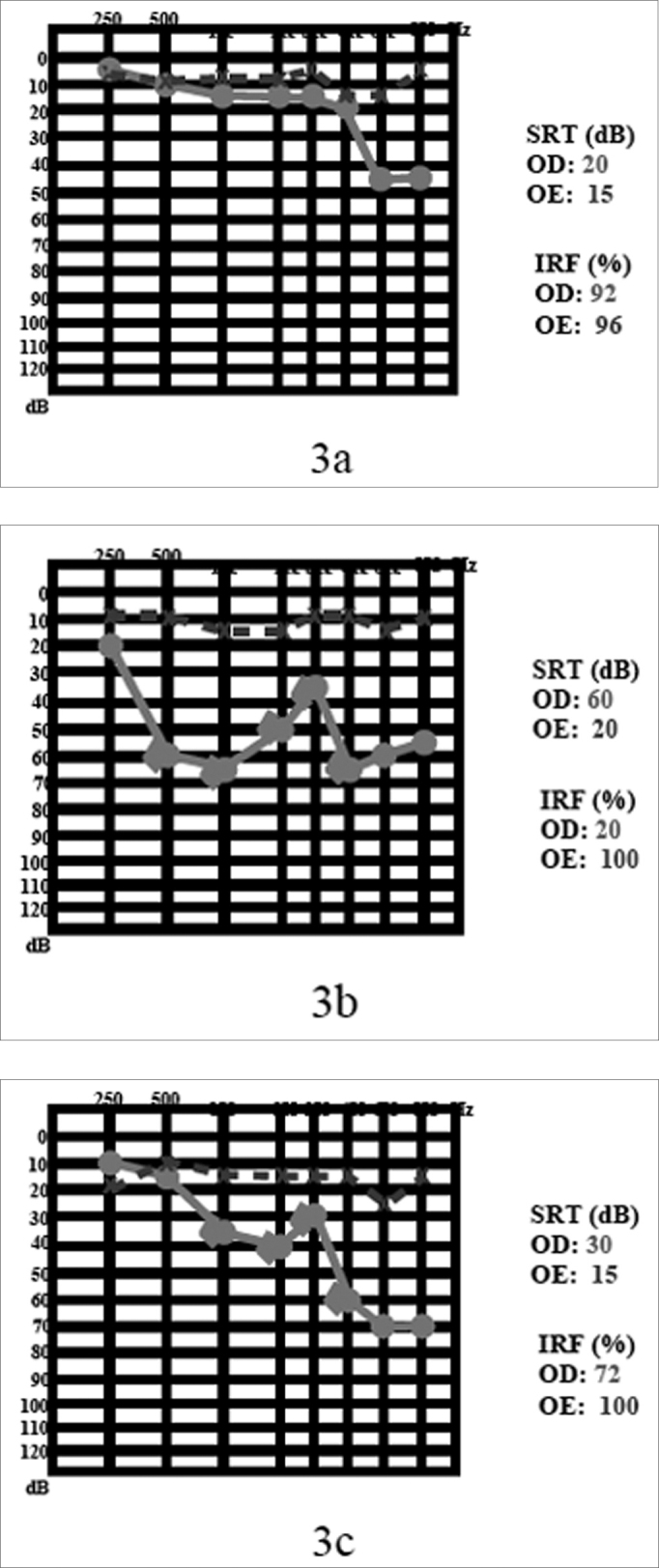
3a) audiogram at the time of diagnosis; 3b) after sudden death; 3c) after 2 years of follow up.

**Figure 4:**
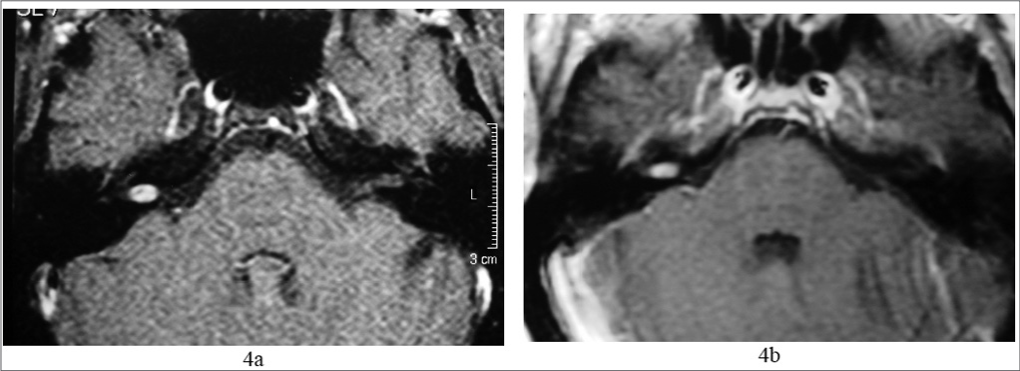
4 a) MRI with gadolinium at the time of diagnosis; 4b) after two years.

She came to us complaining of tinnitus in her right ear for 1 ear, without hearing loss or vertigo. Her physical exam was normal. Her audiogram showed a sensorineural hearing loss (45 dB HL) in 6 and 8kHz on the right side (Figure 3a). MRI showed a tumoral mass of the following dimensions: 0.7 × 0.4 × 0.4 cm in her right acoustic meatus (Figure 4a).

Because of her age, a small tumor size and preserved hearing, in a joint process we decided for the conservative approach, that is to follow her up with audiometric and image exams in series.

After 2 years, the patient had one episode of sudden hearing loss and remained with an SRT of 65 dB and sound discrimination in 20% (Figure 3b). She was treated with prednisone, in the dose of 1mg/kg of body weight, and in the second week of drug treatment her hearing returned to the previous levels (3-c). Control MRI showed a reduction in VS size, shrinking down to 0.6 × 0.4 × 0.4 cm (Figure 4b).

After a new discussion regarding treatment possibilities, we decided to keep the conservative approach.

## DISCUSSION

The natural history behind VS is yet to be completely unveiled, however, it is known that most VS are of slow growth. Schuknecht, histologically reviewing 1400 temporal bones, found an incidence of 0.57% of VS. This is an extremely high value when we compare to the clinically diagnosed cases of VS which is of only 0.001%3. This difference in numbers can indicate that most VS will never become clinically symptomatic, that is, most people with VS will have no consequences from the tumor ever^4^.

Even among diagnosed tumors, most of them (69%) do not grow after diagnosis and tumor involution is known to happen and has been described before6 with figures that reach 16% of the cases that had tumor shrinkage^6^. We unmistakably observed this phenomenon of tumor regression in the cases we report, for objecting to surgery these patients preferred the expectant approach.

Of the small number of patients (31%) that show some degree of tumor growth, it happens very slowly, 70% have a growth at rates of less than 2mm per year6. Many authors confirm the slow growth of VS, presenting rates of 2.1mm per year or less^2^,^6^,^7^,^9^,^10^.

Unfortunately we can not state that all VS will grow slowly, because there is a relevant variation in tumor growth rate when we compare individuals with it. Thus, to follow the growth rate of VS in cases of small tumors during the fist year after diagnosis with image exams in series, allows us to predict tumor development in a patient during a period that varies between one and three years^2^,^6^,^7^.

Treatment of choice for VS still is complete tumor removal. The surgical microscope and modern surgical approaches for the pontine-cerebellar angle have brought about an important reduction in the surgery mortality rate, to less than 1%^2^, and today the goal is not only complete tumor removal, but also the preservation of facial and cochlear nerve function. Despite the high rates of treatment success, there still is the possibility of facial paresis or paralysis or a worsening in postoperative auditory thresholds; and these are the factors that really impact the patient's decision for a conservative approach. Our patient reported in case 1 had a mild worsening in his auditory threshold during this short observation period, only in the high frequencies, thus, the observing approach did not bring him any neurologic risk and he has been able to have what we call a “social hearing” throughout these last five years, which is something not guaranteed by surgery. At any time, if his hearing starts to go down, we can propose to discontinue this observational approach without harm to the patient. Another aspect that must be taken into account in dealing with intracanal VS cases is the improvement in hearing thresholds with the use of steroids after sudden hearing loss installs, and this was clearly seen in the patient presented in case 2. This patient received high doses (1mg/kg/day) and normalized her thresholds and sound discrimination in two weeks. The mechanism by which steroids would work in theses cases of sudden hearing loss in VS patients is still unknown, however the phenomenon has been described before.^10^

Considering the major progress of image exams lately, especially that of MRI, there are a larger number of patients with VS diagnosed earlier on. This means that we are diagnosing more and smaller tumors that cause less symptoms. Considering the natural history of VS, we still have the initial doubt about which treatment modality to choose from: the more aggressive surgical approach or the conservative, waiting and observing approach. Having in mind that, besides the slow growth rate associated with the tumor in most cases, we must also consider the possibilities of sequelae remaining from the surgical approach, which can be worse than the initial symptoms.

The conservative approach is preferred by many authors in a selected group of patients: those with advanced age, minimum symptoms, unfavorable clinical conditions, small tumors, tumors in a single ear or those who do not wish to operate, as long as this does not bring about any neurologic risk.

We must consider that the conservative approach does not mean surgery aversion, but rather an initial follow up in order to see if the tumor the patient harbors is of slow or fast growth. The surgical approach can be considered when there is a fast increase in tumor size seen in a series of image exams, when there is symptoms worsening or even in those patients who first decided for a conservative approach but later decide for a more definitive treatment. We must also stress that audiogram worsening is not statistically associated with tumor growth^11^.

According to Fisch5, the initial conservative approach is worth it because only 12% of the patients included in his research who were initially included in the group for conservative treatment ended up requiring surgery. In these patients, tumor growth rate was much greater when compared to those who did not require surgery, and moreover the initial tumor size was statistically larger.

The second issue at hand when we decide for a conservative approach in cases of VS is whether we are truly bringing any benefit to the patient when we wait on surgery. Tumor observation time should not allow for a worsening in clinical status, thus compromising surgical prognosis. Some studies have compared patients who were submitted to surgery immediately after diagnosis with those who decided for the conservative approach and were later operated, and these did not show statistically significant differences as far as postoperative sequelae are concerned; thus ratifying the conservative approach in cases of VS^6^,^7^,^9^.

## FINAL REMARKS

A conservative approach should be always considered in patients diagnosed with VS, especially in patients with minimum symptoms, advanced age, patients with other clinical issues, small tumors, tumors in one ear only or those patients who do not wish to undergo surgery. We have to base ourselves on the fact that tumor growth rates in the first year after diagnoses predicts tumor behavior in the following years. The conservative approach can not be inflexible, and should be abandoned in light of evidence of fast tumoral growth, worsening in symptoms or even patient wish. A small number of patients who were initially submitted to conservative treatment needed surgery later on. And finally, a conservative approach to VS does not bring greater risks to the patient, since it does not worsen a later surgical prognosis.
